# The Imperial Cancer Research Fund

**Published:** 1907-07-13

**Authors:** 


### The Imperial Cancer Research Fund.

The annual meeting of the general committee of the Imperial Cancer Research Fund may suitably be made the subject of a few general comments. And first may be noted the fact that the meeting was held at Marlborough House and under the chairmanship of the President, H.R.H. the Prince of Wales. The medical profession, and indeed the general public also, have learned from a long and convincing experience that no sound scheme for the alleviation of suffering and for the diminution of disease appeals in vain to the sympathy and practical wisdom of his Royal Highness. The heir to great and stimulating traditions in connection with such appeals, the Prince has ever proved himself worthy of the noble example set by King Edward, and has extended generous and large-hearted recognition and assistance to all efforts "for the relief of man's estate." Nor will it be forgotten that this attitude is associated with an active and sustained interest in such efforts. It is no mere academic approval and patronage, but a gift of personal service, that prompts the general conviction that where his Royal Highness leads, all may rely both upon wisdom in design and thoroughness in method. It is under a guarantee of this order that the Imperial Cancer Research organisation has pursued, and will continue to pursue, its great and responsible task.

A second feature which marked the recent meeting was the intimation of a number of handsome contributions to the fund, without which continuation of the work would be impossible. And here again the efficacy of the President's interest is seen in the announcement of a gift of £1,000 from an anonymous donor. Naturally and inevitably, too, special recognition must be offered to the splendid and munificent donation of £40,000 contributed by Mr. and Mrs. Bischoffsheim. At the very initiation of the fund Mr. Bischoffsheim gave a sum of £5,000, and was, indeed, one of three original donors by whose generosity the commencement of the work was made possible. He has now in the most practical fashion recorded his conviction of the value and promise of what has already been accomplished, and his anticipation of what is further to be gained. The same may be said of other contributions, as, for example, those from certain of the City Companies and Colonial Governments. Welcome as such gifts are to meet the practical necessities of the position, they carry in addition an expression of confidence in the scheme and methods which the Fund has brought into operation. And these, it must be remembered, are framed with a determination to secure scientific thoroughness and accuracy. In the very nature of the case, therefore, the progress of the research must be slow, and from those unaccustomed to the patient and orderly investigation of obscure and complex biological problems, some expressions of impatience might not have been altogether singular. It is thus all the more gratifying to observe that those who are accustomed to deal with practical affairs and with large commercial undertakings, are not less willing than members of the medical profession to demonstrate their confidence in the sure, even though slow, movement of laboratory investigation. If this is stopped, the work of cancer research ceases, and save for the chance of some fortunate and penetrating inspiration, of which there can be no reasonable anticipation, the prospect of unlocking the secret and effecting the cure of malignant disease comes to a compendious end. This aspect of the matter cannot be presented too emphatically or too often. Whatever criticism on points of detail may be advanced—and there is not the slightest desire to restrain such criticism—the broad fact remains that it is in the application of the scientific method to the problem of cancer that the hope of humanity for rescue from a cruel fate at this moment resides. That application is already in force. To sustain and continue it without money is impossible. It is, therefore, to be hoped that the further sum of £13,000 needed to place the Fund in a secure position will not long be lacking.

